# Screening and risk factors of exocrine pancreatic insufficiency in critically ill adult patients receiving enteral nutrition

**DOI:** 10.1186/cc12850

**Published:** 2013-08-07

**Authors:** Sheng Wang, Lijie Ma, Yugang Zhuang, Bojie Jiang, Xiangyu Zhang

**Affiliations:** 1 Department of Critical Care Medicine, Shanghai Tenth People's Hospital, Tongji University, Shanghai 200072, China; 2 Department of Emergency, Shanghai Tenth People's Hospital, Tongji University, Shanghai 200072, China

## Abstract

**Introduction:**

Malnutrition is a frequent problem associated with detrimental clinical outcomes in critically ill patients. To avoid malnutrition, most studies focus on the prevention of inadequate nutrition delivery, whereas little attention is paid to the potential role of exocrine pancreatic insufficiency (EPI). In this trial, we aim to evaluate the prevalence of EPI and identify its potential risk factors in critically ill adult patients without preexisting pancreatic diseases.

**Methods:**

In this prospective cross-sectional study, we recruited 563 adult patients with critical illnesses. All details of the patients were documented, stool samples were collected three to five days following the initiation of enteral nutrition, and faecal elastase 1 (FE-1) concentrations were assayed using an enzyme-linked immunosorbent assay kit. Blood samples were also taken to determine serum amylase and lipase activity.

**Results:**

The percentages of recruited patients with EPI (FE-1 concentration <200 μg/g) and severe EPI (FE-1 concentration <100 μg/g) were 52.2% and 18.3%, respectively. The incidences of steatorrhea were significantly different (*P *< 0.05) among the patients without EPI, with moderate EPI (FE-1 concentration = 100 to 200 μg/g) and severe EPI (FE-1 concentration < 100 μg/g). Both multivariate logistic regression analysis and z-tests indicated that the occurrence of EPI was closely associated with shock, sepsis, diabetes, cardiac arrest, hyperlactacidemia, invasive mechanical ventilation and haemodialysis.

**Conclusions:**

More than 50% of critically ill adult patients without primary pancreatic diseases had EPI, and nearly one-fifth of them had severe EPI. The risk factors for EPI included shock, sepsis, diabetes, cardiac arrest, hyperlactacidemia, invasive mechanical ventilation and haemodialysis.

**Trial registration:**

NCT01753024

## Introduction

It is well-known that malnutrition, a common problem in patients admitted to ICU, has a negative impact on clinical outcomes, such as higher risk of infection and multiple organ dysfunction, prolonged mechanical ventilation and hospital stay, and increased morbidity and mortality [1,2]. Moreover, it has been proposed that the incidence of malnutrition will be more frequent in coming decades because of the increasing number of ICU patients who are older and/or obese and have chronic diseases, which inevitably aggravate stress-related catabolism and negative energy balance [1]. To prevent the occurrence of malnutrition, the vast majority of current studies focus on strategies to improve the efficacy of nutritional support [2,3], as insufficient nutritional delivery has been considered to be a crucial determinant of malnutrition in critically ill patients [4-6].

Malnutrition is also a major consequence and clinical manifestation of exocrine pancreatic insufficiency (EPI), because pancreatic enzymes are essential for the digestion of macronutrients and inadequate release of pancreatic enzymes leads to maldigestion and malabsorption of fat, as well as, to a lesser extent, proteins and carbohydrates [78]. More importantly, pancreatic damage is frequently observed in critically ill patients without primary pancreatic diseases, although the majority of these diseases do not develop into pancreatitis. For example, Manjuck *et al. *[9] reported that the incidences of hyperamylasemia and hyperlipasemia in interdisciplinary ICU patients without prior pancreatic diseases were 32.7% and 40.4%, respectively. Denz *et al. *[10] found that morphological alterations of the pancreas were detected by computed tomography in 35% of ICU patients without preexisting pancreatic diseases and that elevated serum lipase activity was present in 78.8% of critically ill patients. Tribl *et al. *[11] discovered that exocrine pancreatic dysfunction occurred in ICU patients in the absence of significant pancreatic enzyme elevation or histological evidence of pancreatic damage, suggesting that a broad spectrum of pancreatic damage may be present in critically ill patients. Based on the high incidence of pancreatic damage in ICU patients, it is reasonable to speculate that EPI might be another critical contributor to malnutrition in addition to inadequate intake of nutrition in critically ill patients.

Unfortunately, little is known about the prevalence of EPI and its determinants in critically ill patients as well as about the underlying association between malnutrition and EPI. Therefore, we performed the present study to screen for EPI and try to identify the potential risk factors for EPI in critically ill adult patients without primary pancreatic diseases who were receiving early enteral nutrition (EN).

## Materials and methods

### Study population

From December 2011 to November 2012, 1,679 critically ill patients were admitted to the emergency ICU and the general ICU at Shanghai Tenth People's Hospital, Tongji University, and both ICUs were within a fully enclosed medical and surgical mixed setup. The patients who required early EN and were expected to stay in the ICU for at least three days were considered for enrolment into this study. Exclusion criteria were age under 18 years, pregnancy or breastfeeding, and any patients with comorbid preexisting EPI due to pancreatitis, cystic fibrosis, celiac disease, Zollinger-Ellison syndrome, pancreatic and ampullary tumours, or gastrointestinal and pancreatic surgical resections [7,8]. Patients were also excluded if they were prescribed drugs (including somatostatin, aprotinin, valproate and carbamazepine) that may affect the secretion of pancreatic enzymes or if they were accompanied by EN intolerance for three consecutive days, which was defined as the disruption of EN feeding plan due to any of the following reasons: prefeeding gastric residual volume more than 500 ml, aspiration, diarrhoea, severe abdominal distention or gastrointestinal bleeding. A total of 563 patients were finally recruited into this prospective cross-sectional study, and informed consent documents were signed by immediate family members of these patients. The study protocol was performed in accordance with the institutional guidelines for the conduct of research on human beings and approved by the Human Ethics Committee of Shanghai Tenth People's Hospital.

### Study design

Once the enrolled patients were admitted to the ICU, either a nasogastric tube or a nasojejunal tube (Nutricia Pharmaceutical Co, Shanghai, China) was inserted, guided by an electronic gastroscope according to the expected feeding time, and the position of the feeding tube was confirmed by plain abdominal radiographs. EN was initiated at a rate of 25 ml/h within 24 hours of admission, and the infusion rate was increased steadily until the prescribed nutritional requirements were achieved within three days. Tube feeding may be temporarily discontinued because of intolerance of EN but resumed at a lower infusion rate four to six hours later. The formula of the EN diet was an isotonic EN suspension with dietary fibre (Jevity; Abbott, Hoofddorp, the Netherlands) which contained 20 g of protein, 17 g of fat, 70 g of carbohydrates and 525 calories of energy per 500 ml. Daily nutritional requirements were calculated by a dietitian and mainly based on the patient's body mass index (BMI). To achieve quality bowel movements, the bowel motion and sounds of all recruited patients were assessed and documented daily, and lactulose (15 ml; Abbott, Shanghai, China) and rhubarb soda (three tablets; Shanghai Traditional Chinese Pharmaceutical, Shanghai, China) were routinely given three times per day. If no bowel movement occurred on the second day of admission, an itopride hydrochloride tablet (50 mg; Abbott, Tokyo, Japan) was initiated orally three times per day and glycerine enema (110 ml; Shanghai Huangpu Pharmaceutical, Shanghai, China) was used rectally as required. If a patient had no bowel motion within 48 hours after admission, an abdominal X-ray was done to exclude ileus and paraffin oil or vegetable oil (40 ml) was applied orally two times per day. Once diarrhoea was present, these bowel protocols were terminated until bowel movements were absent again.

During the study, the details of each patient at admission, such as age, sex, BMI, diagnosis and Acute Physiology and Chronic Health Evaluation II (APACHE II) score, were collected. The incidences of steatorrhea (loose, frothy, foul-smelling and buoyant stools) and diarrhoea (more than three loose bowel movements per day) following the initiation of EN were also documented. Clinical characteristics that may cause exocrine pancreatic damage, including shock (systolic blood pressure <90 mmHg), tissue hypoxia (serum lactate >2 mmol/L), respiratory failure (partial pressure of oxygen in arterial blood <60 mmHg), anaemia (haemoglobin <80 g/L), obesity (BMI >30 kg/m^2^), hyperbilirubinemia (total bilirubin >17.5 μmol/L), hypertriglyceridemia (> 1.7 mmol/L), diabetes (fasting blood glucose ≥7 mmol/L), sepsis (systemic inflammatory response syndrome plus documented infection), brain injury (due to severe trauma, acute stroke or neurosurgery), cardiac arrest, invasive mechanical ventilation and continuous renal replacement therapy (CRRT) were recorded prospectively. Stool samples were collected three to five days after the onset of EN and frozen at -20°C until analysis, and arterial blood samples were also taken at the time to determine serum amylase and lipase levels.

### Biochemical measurements

The enzymatic activities of serum amylase and lipase were measured using a biochemistry analyser (VITROS 350; Ortho Clinical Diagnostics, Melbourne, Australia) at our clinical laboratory centre. Measurements were repeated using duplicated blood samples, and the normal reference values of serum amylase and lipase were less than 190 U/L and 220 U/L, respectively.

Faecal elastase 1 (FE-1) concentrations in the stool specimens were determined by using an enzyme-linked immunosorbent assay kit (ScheBo BioTech AG, Giessen, Germany) as described elsewhere [12]. Briefly, two monoclonal antibodies against different specific epitopes of human pancreatic elastase 1 were applied, and the antigen-antibody complex was revealed by the addition of peroxidase-streptavidin, which was able to bind the biotin-conjugated second monoclonal antibody. The concentrations of oxidized peroxidase substrate were assayed by using a microplate reader (Infinite 200 PRO; Tecan, Männedorf, Switzerland) at 405 nm with 492 nm as the reference wavelength. Duplicate assays were carried out, and FE-1 concentrations were calculated from the standard curve. The results were expressed as micrograms of FE-1 per gram of stool. Typically, the normal level of FE-1 concentration is no less than 200 μg/g, with a value less than 100 μg/g indicating severe EPI and a value between 100 μg/g and 200 μg/g classified as moderate EPI [13,14].

### Statistical analysis

All data were analysed using SigmaStat 3.5 software (Systat Software Inc, San Jose, CA, USA), and *P *< 0.05 was considered statistically significant. Quantitative variables were expressed as mean ± standard deviation (SD), and differences between groups were assessed by one-way analysis of variance followed by a Tukey test when appropriate. Categorical variables were presented as proportions, and the comparison between groups was evaluated by z-test. The odds ratios (ORs) and 95% confidence intervals (CIs) were estimated by multivariate logistic regression analysis, in which the dependent variable was EPI (FE-1 <200 μg/g), and the independent covariables included shock, anaemia, sepsis, diabetes, obesity, cardiac arrest, respiratory failure, hyperbilirubinemia, brain injury, hyperlactacidemia, hypertriglyceridemia, invasive mechanical ventilation and CRRT.

## Results

**Figure 1:**
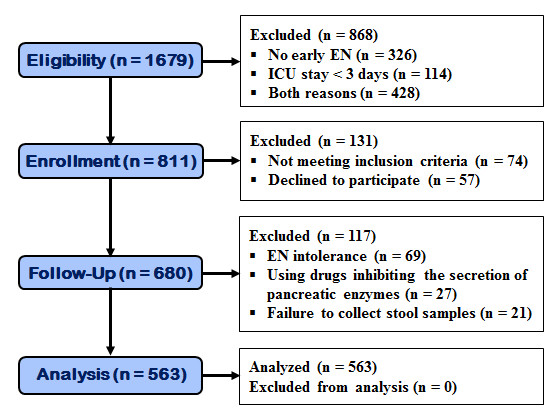
**The CONSORT flow diagram for the study participants**. EN, enteral nutrition.

Of 1,679 critically ill adult patients assessed for eligibility, 811 patients were considered for recruitment into the present study. Seventy-four patients were excluded because they did not meet the inclusion criteria, and another fifty-seven patients were excluded because they refused to participate (Figure 1). Among the 680 patients enrolled into this study, 69, 27 and 21 patients were excluded because of EN intolerance, the application of pancreatic secretion-suppressing drugs and failure to collect stool specimens within three to five days following early EN, respectively (Figure 1). Thus, data collection and analysis were performed in a total of 563 patients.

Among the recruited ICU patients (*N *= 563) without EPI, with moderate EPI or with severe EPI, there were no statistical differences in age, gender, BMI and APACHE II score, but the incidences of steatorrhea and diarrhoea and the levels of serum amylase and lipase were significantly higher in the patients with severe EPI than in those without EPI (Table 1). Moreover, the incidence of steatorrhea in the patients with severe EPI was also higher than that in those with moderate EPI.

**Table 1:** Characteristics of patients with different levels of exocrine pancreatic insufficiency (*N *= 563)^a^

Characteristics	No EPI (*n *= 269)	Moderate EPI (*n *= 191)	Severe EPI (*n *= 103)
Age (years)	40.9 ± 3.4	43.8 ± 4.1	41.7 ± 5.9
Gender (% male)	46.8	43.9	42.4
BMI (kg/m^2^)	25.2 ± 2.6	27.4 ± 3.5	28.1 ± 5.3
APACHE II score	12.7 ± 3.8	16.3 ± 2.9	17.9 ± 4.7
Steatorrhea (%)	2.2	5.3^b^	11.7^b,c^
Diarrhoea (%)	9.7	13.9	21.5^b^
Serum amylase (U/L)	218 ± 51	319 ± 94	494 ± 123^b^
Serum lipase (U/L)	252 ± 67	405 ± 102	576 ± 139^b^
Faecal elastase 1 (μg/g)	276 ± 75	161 ± 42	71 ± 24^b^

^a^APACHE II, Acute Physiology and Chronic Health Evaluation II; BMI, body mass index; EPI, exocrine pancreatic insufficiency. ^b^*P *< 0.05 in comparison with patients without EPI. ^c^*P *< 0.05 in comparison with patients with moderate EPI. Data are presented as mean ± SD or percentage.

**Figure 2:**
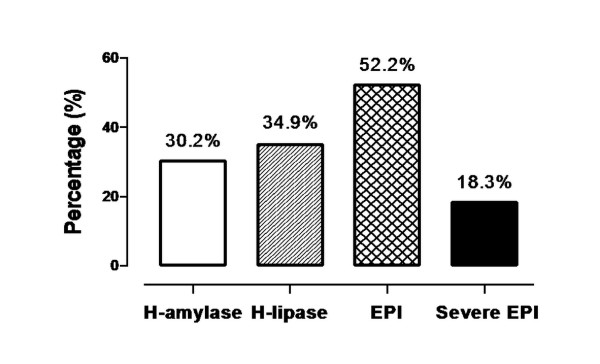
**Occurrence of exocrine pancreatic insufficiency, severe exocrine pancreatic insufficiency, hyperamylasemia and hyperlipasemia in critically ill adult patients**. EPI, exocrine pancreatic insufficiency; H-amylase, hyperamylasemia; H-lipase, hyperlipasemia.

The prevalence of hyperamylasemia (>190 U/L), hyperlipasemia (>220 U/L), EPI (FE-1 concentration <200 μg/g) and severe EPI (FE-1 concentration <100 μg/g) in the enrolled ICU patients is illustrated in Figure 2. Among these data, the highest was EPI (52.2%), followed by hyperlipasemia (34.9%), hyperamylasemia (30.2%) and severe EPI (18.3%).

In the patients with EPI, the percentages of patients with shock, sepsis, diabetes, cardiac arrest, hyperlactacidemia, invasive mechanical ventilation or CRRT were significantly higher than in those without EPI (Table 2). Furthermore, multivariate logistic regression analysis indicated that the occurrence of EPI was directly associated with shock, sepsis, diabetes, cardiac arrest, hyperlactacidemia, invasive mechanical ventilation and CRRT (Table 3).

**Table 2:** Association between exocrine pancreatic insufficiency and clinical characteristics in critically ill adult patients^a^

Characteristics	Number of Patients	EPI (*n *= 294)	None-EPI (*n *= 269)	*P *value
Shock	114	28.9	10.8	<0.001^b^
Anaemia	64	8.8	14.1	0.065
Sepsis	78	17.7	9.7	0.009^b^
Diabetes	121	27.2	15.2	<0.001^b^
Obesity	293	51.0	53.2	0.662
Cardiac arrest	83	21.1	7.8	<0.001^b^
Respiratory failure	173	28.6	33.1	0.287
Hyperbilirubinemia	43	9.2	6.3	0.124
Brain injury	185	35.7	29.7	0.154
Hyperlactacidemia	136	33.0	14.5	<0.001^b^
Hypertriglyceridemia	114	22.8	17.5	0.145
Mechanical ventilation	281	57.1	42.0	<0.001^b^
CRRT	58	13.9	6.3	0.005^b^

^a ^CRRT, continuous renal replacement therapy; EPI, exocrine pancreatic insufficiency. ^b^*P *< 0.05 compared with patients without EPI based on z-test. Data are presented as the percentage of the patients with relevant clinical characteristics.

**Table 3:** Multivariate logistic regression analyses of exocrine pancreatic insufficiency correlated with clinical characteristics^a^

	EPI (FE-1 <200 μg/g)		
	
Characteristics	OR	95% CI	*P *value
Shock	2.65	1.68 to 4.18	<0.001^b^
Anaemia	1.48	1.23 to 1.77	0.301
Sepsis	1.83	1.35 to 2.90	0.014^b^
Diabetes	1.74	1.14 to 2.66	0.010^b^
Obesity	1.51	1.08 to 2.11	0.413
Cardiac arrest	2.93	1.72 to 4.98	<0.001^b^
Respiratory failure	1.05	0.74 to 1.51	0.774
Hyperbilirubinemia	1.26	1.18 to 2.07	0.321
Brain injury	1.18	0.52 to 2.66	0.699
Hyperlactacidemia	2.38	1.58 to 3.61	<0.001^b^
Hypertriglyceridemia	1.11	0.74 to 1.68	0.604
Mechanical ventilation	2.62	1.86 to 3.69	<0.001^b^
CRRT	1.22	1.09 to 2.78	0.026^b^

^a^CI, confidence interval; CRRT, continuous renal replacement therapy; EPI, exocrine pancreatic insufficiency; OR, odds ratio. ^b^*P *< 0.05, estimated by multiple logistic regression analysis.

## Discussion

As measured by FE-1 concentration, EPI occurred in 52.2% of critically ill adult patients without preexisting pancreatic diseases, and nearly one-fifth of them could be diagnosed as having severe EPI. To the best of our knowledge, this trial is the first large-scale investigation to evaluate the prevalence of EPI and severe EPI in a population with critical illness. The results also demonstrated that the occurrence of EPI was closely correlated to clinical characteristics such as shock, sepsis, diabetes, cardiac arrest, hyperlactacidemia, invasive mechanical ventilation and CRRT.

In this study, FE-1 concentration was selected to assess the occurrence and extent of EPI, because FE-1 determination with a cutoff less than 200 μg/g has been shown to have high sensitivity and specificity for the detection of moderate to severe EPI in comparison with the "gold standard" for the evaluation of exocrine pancreatic secretion, the secretin-caerulein test [15,16]. Moreover, faecal concentration of elastase 1 was reported to be significantly correlated with the amount of this enzyme secreted by the exocrine pancreas [15,17], and only one, single, small stool collected at random is sufficient to evaluate exocrine pancreatic status by FE-1 assay [18]. Besides, the variation of FE-1 concentration in stool samples is very limited because of the application of human-specific monoclonal antibodies against pancreatic elastase 1 and FE-1's resistance against intestinal degradation [19]. Elastase 1 in the stool specimens is highly stable, and this test is noninvasive and more easily applied in various clinical situations [20], which is particularly important for critically ill patients in the ICU.

Brydon *et al. *[21] previously found that elastase 1 can be diluted or concentrated in variable degrees during the intestinal passage, and drugs such as prokinetic and morphine-derived agents were frequently prescribed in ICU, which would undoubtedly affect the motility of the gastrointestinal tract and thus confound the results of FE-1 concentration. However, the primary endpoint of our present study was to screen for EPI rather than to accurately diagnose EPI, and FE-1 assay has been known to be the preferred pancreatic function test for the screening of EPI up to now [16]. Furthermore, as a typical clinical symptom of EPI, the incidence of steatorrhea was significantly different among the patients without EPI compared with moderate or severe EPI in this study, suggesting that FE-1 assay was a reliable method to screen for EPI. In addition, early EN, Jevity (EN suspension with dietary fibre) and tube-feeding were chosen as the enteral feeding time, formula and pathway, respectively, in our research protocols. Although we did not determine whether these factors affected the results of FE-1 concentration, it is unlikely that these factors would impact the assessment of EPI, as the same extent of influence on FE-1 concentration was applied among all enrolled patients.

Previous studies have reported that the incidence of elevated serum amylase and lipase activity in critically ill patients without preexisting pancreatic diseases ranged from 14% to 80% [9-11,22,23]. In our present study, the proportions of critically ill adult patients with hyperamylasemia and hyperlipasemia were 30.2% and 34.9% respectively, indicating that the incidence of pancreatic damage in ICU patients is around 30% to 40%. Interestingly, the occurrence of EPI in critically ill adult patients, as defined by less than 200 μg/g FE-1 concentration, was more than 50%. This result was comparable to the data reported in a recent study by Senkal *et al. *[24] and suggested that the occurrence of EPI was much higher than that of pancreatic damage in critically ill patients. Moreover, the huge gap between the occurrence of EPI and pancreatic damage indicated that elevated serum pancreatic enzymes may not be an ideal indicator for the assessment of EPI. This notion was also supported by Tribl *et al. *[11], who disclosed that exocrine pancreatic dysfunction existed without the elevation of serum pancreatic enzymes or histological evidence of pancreatic damage.

With respect to the potential risk factors for EPI in ICU adult patients receiving early EN, our results from both multivariate logistic regression analysis and z-test simultaneously demonstrated that shock, sepsis, diabetes, cardiac arrest, hyperlactacidemia, invasive mechanical ventilation and CRRT were closely associated with the occurrence of EPI. Indeed, many previous investigations have provided direct or indirect evidence of the association between EPI and these clinical characteristics. For example, acute necrotizing pancreatitis has been reported in patients with shock, even in the absence of characteristic clinical and laboratory signs of acute pancreatitis [25]. The impairment of exocrine pancreatic function was found in critically ill patients with sepsis, and the extent of exocrine pancreatic dysfunction was intimately related to the severity of sepsis [26]. The prevalence of EPI in type 1 diabetes mellitus ranged from 45% to 74%, and about 28% to 36% of patients with type 2 diabetes mellitus had EPI [27]. In addition, splanchnic hypoperfusion, mechanical ventilation and CRRT have also been suggested as risk factors for pancreatic damage in critically ill patients [9,11,23,28]. Surprisingly, anaemia, obesity, hypertriglyceridemia, hyperbilirubinemia and brain injury due to severe trauma, acute stroke or neurosurgery have also been suggested as risk factors for pancreatic damage in ICU patients [23,24,29,30]; however, no statistical differences were found in our study. The causes are currently unknown but might be attributable to different study populations and research protocols.

It has been reported that malnutrition occurred in 38% to 88% of ICU patients, which was associated with detrimental clinical outcomes [2]. To provide adequate EN, current studies are focused mostly on the choice of delivery timing, formula selection and route of administration [4]. Little attention has been paid to the potential contribution of EPI to malnutrition. In this study, we found that EPI was present in more than 50% of critically ill adult patients without primary pancreatic diseases and nearly 20% of them had severe EPI. If the patients with preexisting EPI had also been included in this study, the proportion of critically ill adult patients with EPI should have been even higher. Hence, the status of exocrine pancreatic function in ICU patients should be considered seriously when the intention is to prevent undernourishment through EN, particularly for those patients with severe EPI or those who have risk factors of EPI. A number of causes other than EPI may also contribute to malnutrition, however, such as inadequate or unbalanced diet, reduced absorption of nutrients and increased energy consumption [31]. Moreover, Deane *et al. *[32] recently reported that absorption at the luminal surface is impaired and small luminal motility may affect lipid absorption in critically ill patients. Therefore, improved digestion of nutrients by pancreatic enzyme supplementation cannot guarantee that the occurrence of malabsorption and malnutrition will be avoided. Further studies designed to investigate the effects of pancreatic enzyme supplementation on malnutrition in critically ill patients may help to elucidate the underlying association between EPI and malnutrition.

Although our observational study has shown a high prevalence of EPI and disclosed several risk factors for EPI in a large cohort of heterogeneous critically ill patients, a few limitations of the current report must be mentioned. First, the assessment of steatorrheawas based on clinical observation, but not 72-hour faecal fat quantification, the gold standard for the diagnosis of steatorrhea, although the latter has well-known handicaps limiting its clinical applicability [20]. Second, even if elastase 1 has excellent resistance against intestinal degradation [19], the degradation of elastase 1 during the storage of stool samples might potentially have affected the results of FE-1 concentration, as no control stool samples were used to determine the historical reference ranges of FE-1 concentration in this study. Third, EPI was defined as a cutoff concentration of FE-1 less than 200 μg/g in the present study, which means that continuous variables were converted into categorical variables to divide patients into different groups; thus, this procedure had inherent limitations that may result in lost information and reduced power of statistical tests [33].

## Conclusion

EPI, as measured by FE-1 concentration, was present in more than 50% of critically ill adult patients without primary pancreatic diseases, and almost 20% of them could be diagnosed as having severe EPI. This finding suggests that EPI might be another critical contributor to malnutrition in addition to insufficient nutritional delivery in ICU patients, especially in patients with severe EPI or risk factors for EPI, which consist of shock, sepsis, diabetes, cardiac arrest, hyperlactacidemia, invasive mechanical ventilation and haemodialysis.

## Key messages

• As measured by FE-1 concentration, EPI occurred in 52.2% of critically ill adult patients without preexisting pancreatic diseases, and nearly one-fifth of them could be diagnosed as having severe EPI.

• The occurrence of EPI in critically ill adult patients was closely correlated to clinical characteristics such as shock, sepsis, diabetes, cardiac arrest, hyperlactacidemia, invasive mechanical ventilation and CRRT.

• The status of exocrine pancreatic function in critically ill patients should be considered seriously when the intention is to prevent undernourishment through EN, especially for patients with severe EPI or who have risk factors for EPI.

## Abbreviations

APACHE II: Acute Physiology and Chronic Health Evaluation II; BMI: Body mass index; CI: Confidence interval; CRRT: Continuous renal replacement therapy; EN: Enteral nutrition; EPI: Exocrine pancreatic insufficiency; FE-1: Faecal elastase 1; OR: Odds ratio; SD: Standard deviation.

## Competing interests

The authors declare that they have no competing interests.

## Authors' contributions

SW and XYZ were responsible for the conception and design of the study, data interpretation and drafting the manuscript. LJM, YGZ and BJJ participated in the design of the study and the acquisition and analysis of data. SW had primary responsibility for the final content. All authors read and approved the final manuscript.
